# Rare Presentation of Cardiac Tamponade in a Patient With Subclinical Hypothyroidism

**DOI:** 10.7759/cureus.12286

**Published:** 2020-12-25

**Authors:** Muhammad Usman Almani, Muhammad Usman, Abdul Wahab Arif, Muhammad Talha Ayub, Noor Fatima

**Affiliations:** 1 Internal Medicine, John H. Stroger, Jr. Hospital of Cook County, Chicago, USA; 2 Internal Medicine, Cook County Health, Chicago, USA; 3 Cardiovascular Medicine, Rush University Medical Center, Chicago, USA; 4 Internal Medicine, Nishtar Medical University, Multan, PAK

**Keywords:** cardiac tamponade, hypothyroid pericardial effusion, subclinical hypothyroidism

## Abstract

Cardiovascular effects of hypothyroidism include bradycardia, diastolic hypertension, atrial fibrillation, prolonged QT interval leading to torsades de pointes, varying degrees of AV block, accelerated coronary artery disease, and pericardial effusion. Cardiac tamponade is rare in patients with hypothyroidism because of pericardial distensibility and slow accumulation of fluid. The amount and rate of accumulation of pericardial effusion are related to the severity of hypothyroidism. Though rare, significant pericardial effusion can be a manifestation of subclinical hypothyroidism.

## Introduction

Cardiovascular effects of hypothyroidism include bradycardia, diastolic hypertension, atrial fibrillation, prolonged QT interval leading to torsades de pointes, varying degrees of atrioventricular (AV) block, accelerated coronary artery disease, and pericardial effusion [1]. The amount and rate of accumulation of pericardial effusion are related to the severity of hypothyroidism [2]. Severe hypothyroid states have shown to cause large effusions leading to tamponade physiology. Mild and subclinical hypothyroidism-associated pericardial effusions have rarely been reported to cause cardiac tamponade. We aim to report a case of pericardial tamponade associated with subclinical hypothyroidism.

## Case presentation

**Figure 1:**
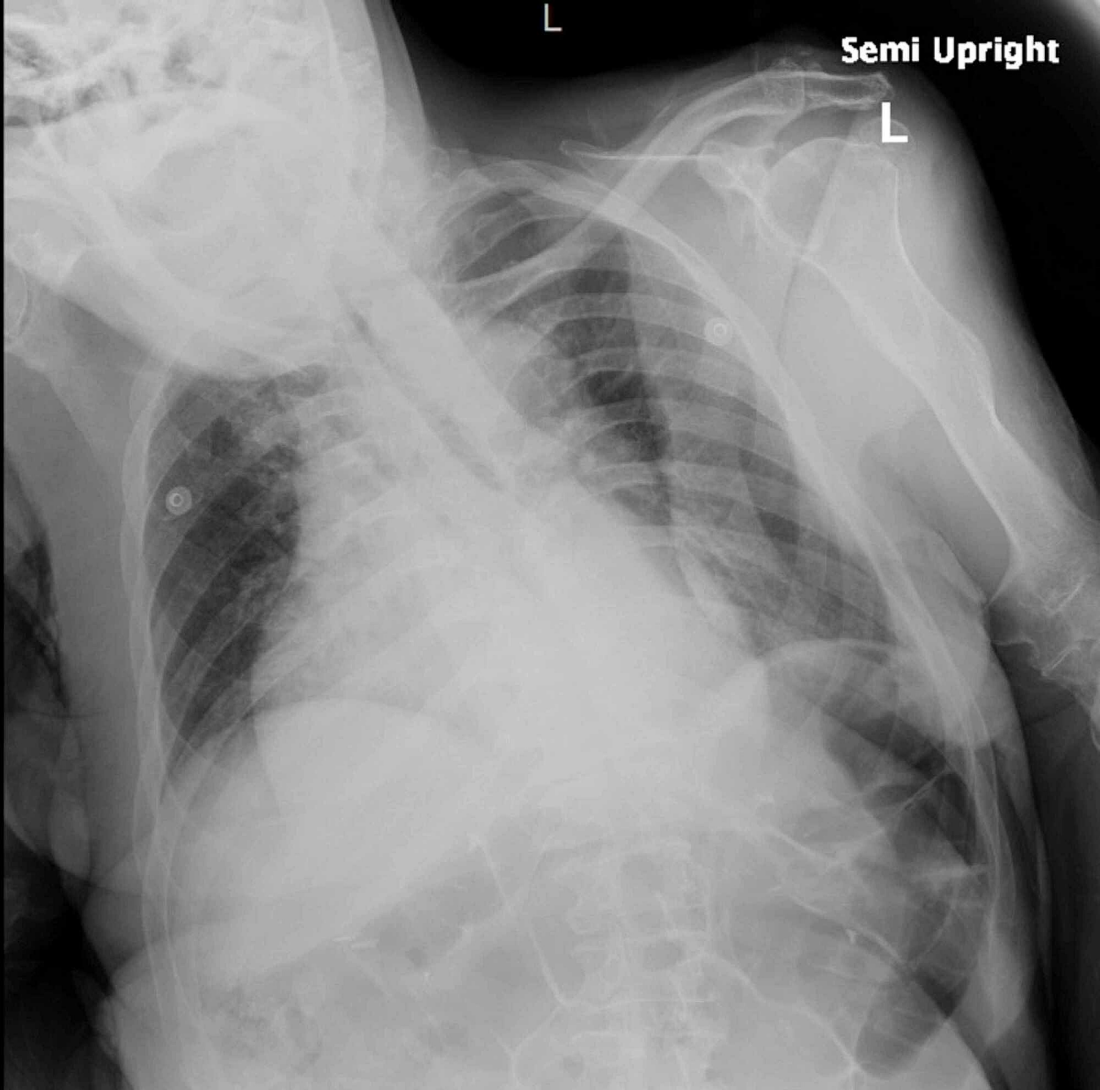
Chest x-ray: enlarged cardiac silhouette

**Figure 2:**
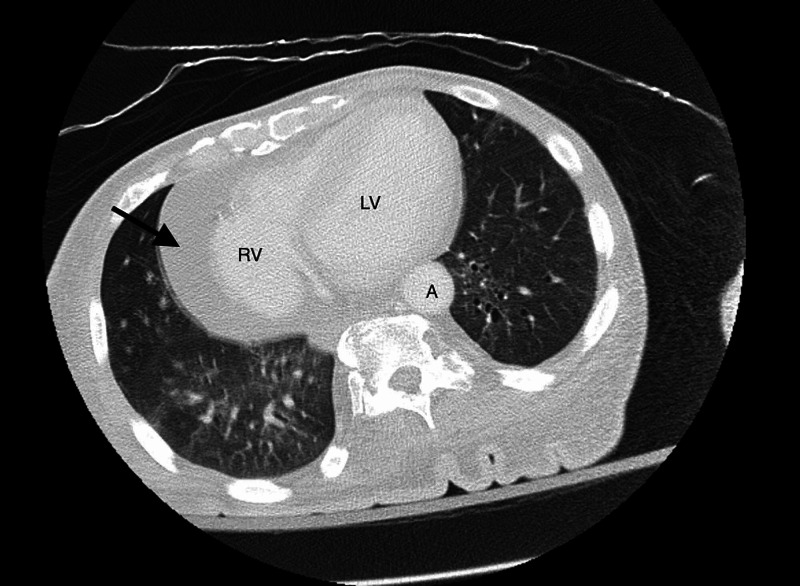
CT chest: moderate- to large-sized pericardial effusion Black arrow showing pericardial effusion; RV: right ventricle; LV: left ventricle; A: aorta; CT: computed tomography.

**Figure 3:**
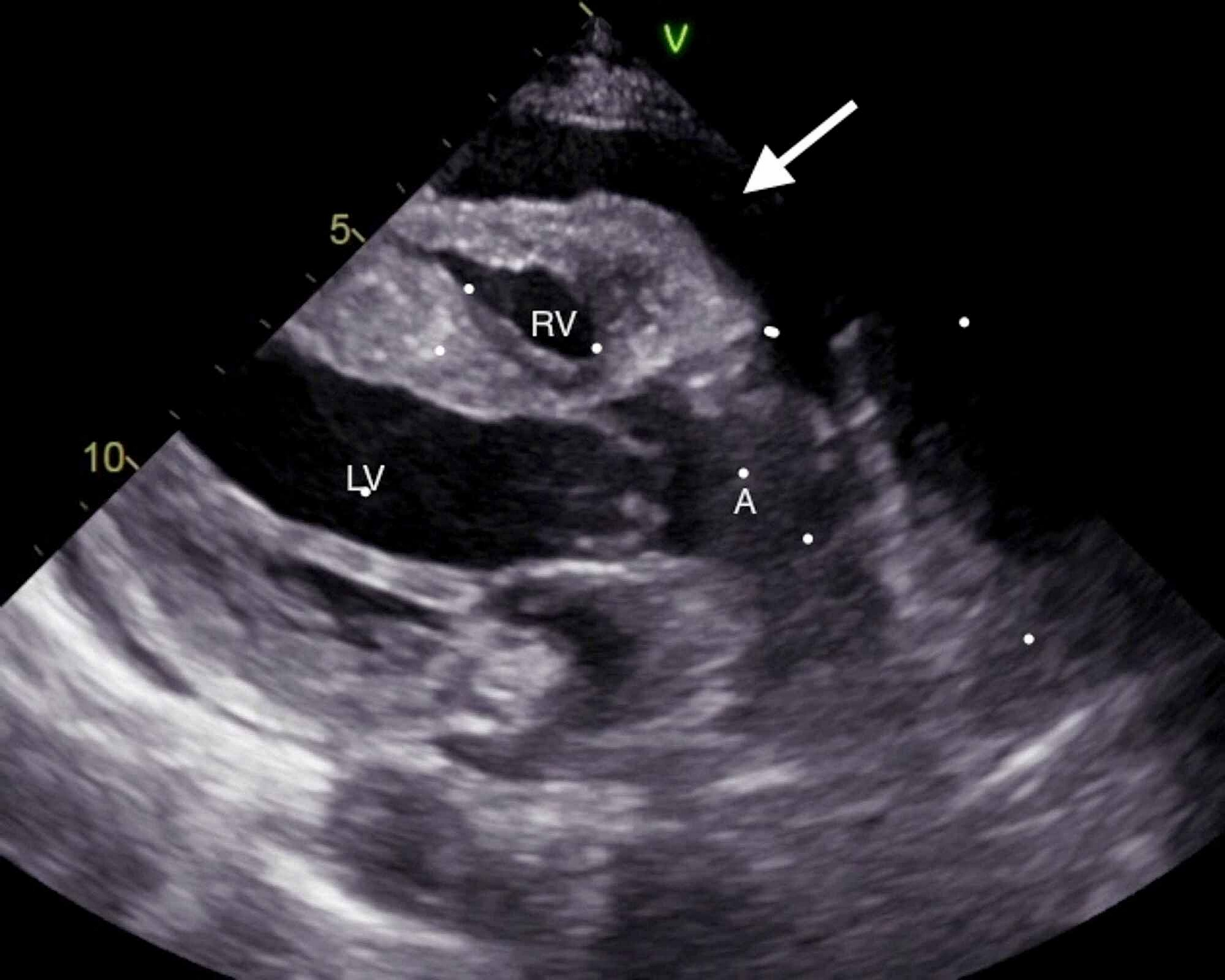
TTE (long para-sternal axis view): anterior pericardial effusion White arrow showing pericardial effusion compressing the right ventricle; TTE: transthoracic echocardiogram; RV: right ventricle, LV: left ventricle; A: aortic root.

A 66-year-old lady with past medical history of Down syndrome, cerebrovascular accident with residual right-sided weakness, vascular dementia, and subclinical hypothyroidism presented with altered mental status for one week. She was noted to have a blood pressure 128/70 mmHg, heart rate 97 beats/min, respiratory rate 18 breaths/min, temperature 97°F. Physical exam was remarkable for Glasgow coma scale score 13, muffled heart sounds. Electrocardiogram (EKG) showed sinus tachycardia with occasional premature ventricular complexes. Chest x-ray revealed large cardiac silhouette (Figure 1). Labs were pertinent for sodium of 139 mEq/L, creatinine of 0.7 mg/dL, bacteriuria and pyuria on urinalysis, macrocytosis without anemia, and thyroid-stimulating hormone (TSH) of 10 µU/mL with free T4 of 1.23 ng/dL. Anti-thyroid peroxidase antibodies and anti-nuclear antibodies were not detected. Respiratory viral panel was negative, and the patient did not report preceding upper respiratory infection symptoms. Computed tomography (CT) scan of chest showed moderate- to large-sized pericardial effusion (Figure 2). CT brain was performed to rule out central cause of altered mental status that was unremarkable except for chronic lacunar infarct. Urine and blood cultures were sent, and the patient was started on PO (per os) levofloxacin for empiric treatment of urinary tract infection (UTI). Transthoracic echocardiography was done to evaluate for pericardial disease that revealed large free-flowing pericardial effusion measuring at least 2.5 cm adjacent to right ventricle (RV) with right atrium (RA) and RV collapse for more than 50% of the cardiac cycle as well as abnormal interventricular septal motion supporting early tamponade physiology (Figure 3). The patient was transferred to cardiac care unit (CCU) for further management. In the meantime, urine culture showed > 100,000 colony-forming units of Escherichia coli sensitive to levofloxacin. The following day she became hypotensive to 92/51 mmHg with pulsus paradoxus and muffled heart sounds. Cardiothoracic surgery was consulted, and the primary recommendation was urgent surgical pericardial drainage. Subsequently, subxiphoid pericardial window was created yielding 230 ml of pink-colored pericardial fluid leading to hemodynamic stability. Fluid analysis showed three white blood cells/µL, total protein of 3.4 g/dL, glucose of 97 mg/dL, and lactate dehydrogenase (LDH) of 185 U/L. No bacterial growth was seen in pericardial fluid, and pericardial biopsy showed fibro-adipose tissue with increased capillary blood vessels. No malignant cells were identified on fluid cytology. The patient returned to her baseline cognition with continued thyroxine supplementation and treatment of urosepsis. Pericardial Blake drain was removed after six days.

The patient was discharged home with 112 mcg levothyroxine, aspirin 81 mg, atorvastatin 40 mg, folate, and cyanocobalamin supplementation. On follow-up clinic visits, TSH and free thyroxine (FT4) normalized with increased thyroxine dose, and she continued to improve clinically.

## Discussion

Cardiac tamponade is usually a consequence of increased pericardial pressure with accumulation of pericardial effusion. Pericardial effusion may be caused by acute pericarditis, tumor, uremia, hypothyroidism, trauma, cardiac surgery, or other inflammatory/noninflammatory conditions [3]. Hypothyroidism is associated with low plasma volume, reduced rate of albumin synthesis and catabolism, increased capillary permeability leading to the extravasation of intravascular proteins, and a longer mean transit time through extravascular spaces leading to an increase in the extravascular mass of albumin [4].

Literature suggests incidence of pericardial effusion in hypothyroidism to be 3%-6%, with effusion size comparable to degree of hypothyroidism [2]. Cardiac tamponade is rare in patients with hypothyroidism because of pericardial distensibility and slow accumulation of fluid leading to significant pericardial fluid collection without hemodynamic compromise [3].

Provocative factors possibly responsible for the formation of massive pericardial effusion leading to cardiac tamponade in hypothyroidism include infection, trauma, spontaneous pericardial hemorrhage, thyroid therapy, and effect of abdominal paracentesis [5]. Down syndrome is often associated with hypothyroidism, and pericardial effusion may be the first presentation, which usually responds to thyroxine therapy [6]. Although there is no sufficient data to suggest that Down syndrome can lead to pericardial effusion independent of hypothyroidism, this possibility cannot be ruled out.

Subclinical hypothyroidism leading to pericardial tamponade has rarely been reported in the literature. Table 1 summarizes the thyroid profile of patients with subclinical hypothyroidism reported in literature who presented with massive pericardial effusion with/without cardiac tamponade.

**Table 1:** Thyroid profile of patients with subclinical hypothyroidism reported in literature who presented with massive pericardial effusion with/without cardiac tamponade UTI: Urinary tract infection; TSH: thyroid-stimulating hormone.

Author	Sex	Age	TSH (µU/mL)	Free T4 (ng/dL)	Precipitating factor	Cardiac tamponade identified
Papakonstantinou et al. [7]	70	F	30.25	0.81	UTI	No
Setty et al. [8]	50	F	10.17	Not reported	None identified	Yes

Achieving a euthyroid state is the definitive treatment, and in most cases it is the only treatment that is needed for pericardial effusions due to hypothyroidism. However, for pericardial effusion progressing to cardiac tamponade with hemodynamic instability, drainage via either pericardiocentesis or surgical window becomes necessary [9]. Whether thyroxine replacement therapy be targeted to the rate of accumulation of effusion is an interesting research question.

## Conclusions

This case aims to highlight the possibility of cardiac tamponade in the setting of subclinical hypothyroidism. High clinical suspicion should be used for diagnosis. Treatment should also be directed toward any identified precipitating cause as described in the discussion. Serial echocardiographic monitoring of hypothyroidism-associated pericardial effusion can help detect at risk cases.
